# Apparent Breed Predilection for Equid Herpesvirus-1-Associated Myeloencephalopathy (EHM) in a Multiple-Breed Herd

**DOI:** 10.3390/pathogens10050537

**Published:** 2021-04-29

**Authors:** Eva Klouth, Yury Zablotski, Lutz S. Goehring

**Affiliations:** 1 Equine Hospital Center for Clinical Veterinary Medicine, Faculty of Veterinary Medicine Ludwig-Maximilians University, 80539 Munich, Germany; Goehring@pferd.vetmed.uni-muenchen.de; 2 Center for Clinical Veterinary Medicine, Faculty of Veterinary Medicine Ludwig-Maximilians University, 80539 Munich, Germany; Y.Zablotski@med.vetmed.uni-muenchen.de

**Keywords:** EHV-1, risk factors, warmblood, Fjord horse, equine, horses

## Abstract

Equid herpesvirus type 1 (EHV-1) causes several outbreaks of abortion and/or equid herpesvirus-associated myeloencephalopathy (EHM) worldwide each year. EHM is of great concern, as permanent neurological gait anomalies can leave a horse unfit for future use. The study assesses the risk factors associated with the occurrence of EHM. During an unmitigated outbreak, 141 adult horses/ponies of several distinct breeds were evaluated—using multiple Bayesian logistic regression calculating the odds ratios for breed, age, and sex. In total, 33 of the 141 horses showed signs of EHM. Fjord horses and warmblood horses were overrepresented among those developing EHM. The pony breeds, Welsh and Shetland ponies, were underrepresented. In addition, age and sex were not associated with the risk for EHM. The main limitation was that it was a retrospective analysis with some flaws of documentation. It can be concluded that breed was a significant risk factor for developing EHM during this outbreak.

## 1. Introduction

Equid herpesvirus-1 (EHV-1) is a worldwide cause of contagious infections in horses and ponies. As a respiratory tract infection can be followed by cell-associated viremia in peripheral blood mononuclear cells (PBMCs), EHV-1 infection may lead to secondary complications such as EHV-1-associated myeloencephalopathy (EHM), abortion, neonatal death, and chorioidoretinopathy. Details of pathogenesis are yet to be uncovered; however, it involves (restricted) endothelial cell infection of the eye, the pregnant uterus, and the spinal cord. EHM is the result of random and multi-focal acute vasculitis and thrombotic infarctions of the spinal cord (micro)vasculature. Depending on the extent of infarction, clinical signs will include ataxia, dysmetria, and loss of strength in all four limbs. With a further increase in the quantity and quality of infarctive events, the horse will become recumbent [1]. While all equidae are susceptible to primary (respiratory tract) EHV-1 infection, the risk of developing EHM is perceived to be unevenly distributed between different breeds or age groups, and between male and female animals [1,2,3]. EHM has been recognized as a multi-factorial disease. The virus and the magnitude and duration of viremia are frequently discussed, but the risk factors breed, age, and sex are not always considered while analyzing final outbreak numbers [3,4,5].

Here, we describe the course of a fairly unmitigated EHV-1 infection outbreak in a large, (EHV-1) unvaccinated (except 1 animal) multiple-breed herd. The purpose of this study was to investigate retrospectively whether predilection for breed, age, or sex influenced the outcome (EHM) during this outbreak.

## 2. Results

**Figure 1:**
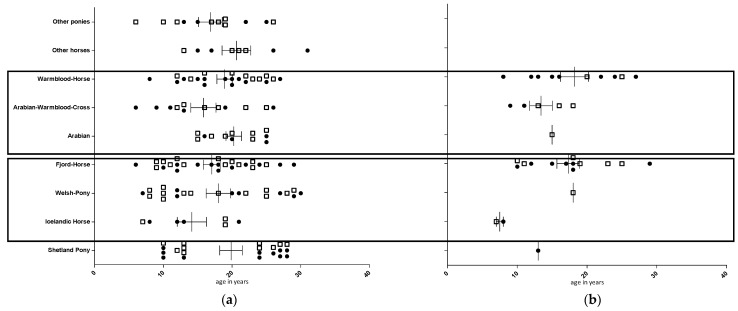
Herd distribution according to breed (*y*-axis), age in years (*x*-axis), and sex (female: open squares; male (castrated): closed circles). Panel (**a**): total group at the beginning of the EHV-1 outbreak. Panel (**b**): EHM cases. Two frames indicate animals housed by night in the same stable building.

On 23 December 2016, a gelding was noticed with dysuria. Shortly afterwards, it became recumbent and was euthanized. A postmortem-collected nasal swab of this horse was submitted to a commercial laboratory for EHV-1 DNA testing using quantitative (q)PCR analysis. The laboratory reported a positive result, but a further differentiation into D- or N-subtype was not pursued. This horse was considered the index case (IC) of this outbreak. A few days later, a second horse was euthanized due to recumbency and transported to the state diagnostic laboratory (LGL Erlangen, Bavaria, Germany) for postmortem examination. Spinal cord tissue was found to be qPCR-positive for EHV-1 DNA. At that time, quarantine was announced for the facility. Quarantine was lifted by the end of March 2017. During this time, horses/ponies were not allowed to enter or leave the premises (except for body disposal), and horse owners were asked to tend to their own horses only. Other mitigating efforts such as barrier precautions, separate small-group arrangements, setting up of an isolation facility, or special personnel instructions were not implemented. Importantly, the daily routine of the combined turn-out of all horses/ponies was maintained during this entire period. Of the 141 animals, up to 50 (35%) were found with a fever for at least 1 day. A total of 33 animals developed EHM (Figure 1b, Table 1). Twenty-five animals were categorized with EHM grade I severity and 8 animals with EHM grade II severity. Immediately following the positive qPCR results in the second EHM case, each animal received a single dose of an avipox-based immunomodulator (Zylexis^®^, Zoetis, Berlin, Germany). Any animal detected with a fever received variable doses of flunixin meglumine or meloxicam (both from Boehringer Ingelheim, Ingelheim, Germany) for the duration of a fever episode. About 10 animals with high and prolonged fevers were treated with dexamethasone (once daily, for 2–4 days, no dose higher than 0.05 mg/kg). Unfortunately, any further detailed documentation regarding the specific animal and the dosage and duration of treatment is lacking. 

Only horses with a defined genetical background (breed) and group size >6 were included in the multiple logistic regression (*n* = 121) analysis for “breed.” This excluded the two groups “other ponies” and “other horses.” Results of the multiple logistic regression show a strong correlation between breed and EHM (Table 2). Fjord horses were the group with the highest number of EHM cases and served as the reference breed. Warmblood horses did not differ (OR = 1.10; CI = 0.37–3.29; *p* = 0.866) compared to Fjord horses. However, Shetland ponies (OR = 0.09; CI = 0.02–0.55; *p* = 0.008) and Welsh ponies (OR = 0.09; CI = 0.01–0.52; *p* = 0.007) apparently had a lower risk of developing EHM. Pairwise comparison of all breeds via post hocs showed that Fjord and warmblood horses had an increased risk for EHM compared to Shetland and Welsh ponies. EHM grade II severity had only 8 observations; however, all but one of the observations (7-year-old Icelandic mare) were either in the Fjord (*n* = 4) or in the warmblood (*n* = 3) group.

EHM was evenly distributed among age. Furthermore, age did not influence the development of either EHM-I or EHM-II during this outbreak. As a total of 14 male and 11 female animals showed EHM-I signs and 6 geldings and 4 mares developed grade II EHM, sex had no influence on the development of EHM in this outbreak (Table 3). As a side note, the only EHV-1-vaccinated horse, a 12-year-old Icelandic horse gelding, had a fever for two days but no signs of EHM. 

**Figure 2:**
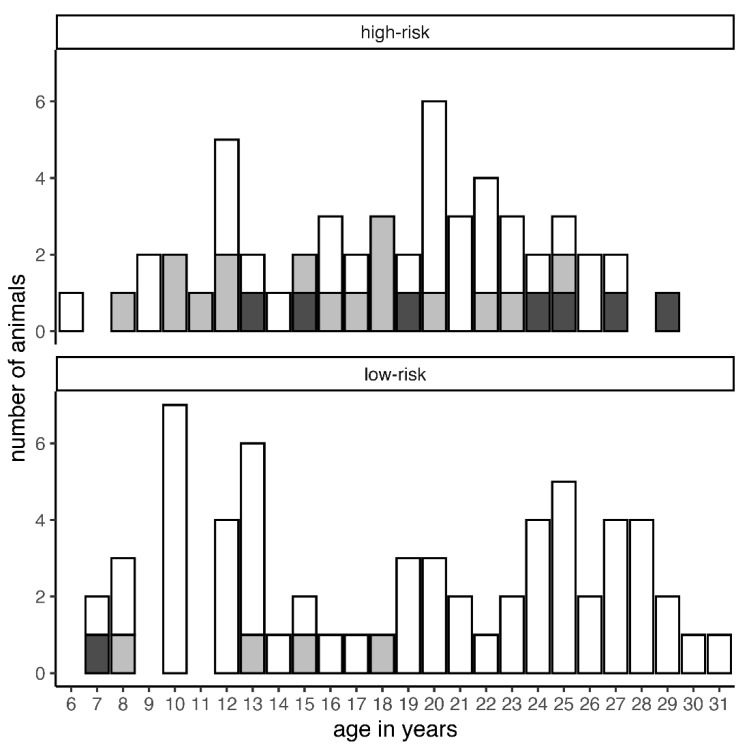
Separation of a total of 115 animals into high-risk and low-risk breeds with age on the *x*-axis: blank = unaffected; light gray = EHM grade I; dark gray = EHM grade II.

We re-grouped breeds into “high-risk EHM” and “low-risk EHM” based on our observations during this outbreak, and on outbreak reports of single (or predominant) breed outbreaks published in peer-reviewed literature. The purpose was to control for other factors. We considered as high-risk EHM breeds Fjord and Warmblood horses and, based on the literature, Draft horses and Quarter horses [2,6]. Low-risk EHM breeds included Shetland ponies, Icelandic horses, Welsh ponies, Haflinger horses, and Arabians (the latter three because currently there are no reports of natural EHM outbreaks on single-breed operations in the peer-reviewed scientific literature). Only horses with a defined genetical background and no crossbreeds participated in this evaluation. The number of horses available for this assessment was reduced to *n* = 115 (Figure 2). This allowed us to include the few other members of defined breeds, while crossbred horses or unknown breeds were excluded. The only observation was that EHM cases in the low-risk group were found in the lower (=younger) arithmetic half (Figure 2).

## 3. Discussion

There is ample discussion on how viral factors of EHV-1 influence the course of an EHV-1 outbreak and their role in causing EHM [6]. There are few reports focusing on breed, age, and sex as risk factors, but there is an ongoing debate on EHV-1 vaccine efficacy and EHV-1-related disease prevention. 

Early outbreak descriptions focus on single-breed operations, on predominantly female populations on stud farms, or on outbreaks at training facilities with a relatively young or adolescent horse population [7,8,9,10]. Two reports describe natural disease outbreaks in mixed equine populations [11,12]; however, comparing strata of subpopulations lacked statistical power, or the data were affected by the confounding factors of “location on the premises” or “intervention during the outbreak.” Here, we present the data of an EHV-1 infection outbreak with very little intervention and mitigation, which resulted in an overall 23% incidence rate of EHM cases. This outbreak started during early winter (Northern Hemisphere), while the period between winter and early spring is typically the season with the most EHM outbreaks. The premises were quarantined for 3 months, which is unusually long for an EHV-1 outbreak. Explanations are likely the size and lack of mitigating efforts, which in general should include testing aimed at the identification of shedding animals followed by separation from the uninfected group of animals. This outbreak is unique because of the total number of horses/ponies that must have been exposed to the virus due to uninhibited transmission and the heterogeneity of the population, yet capable of being divided into sizable groups of distinct breeds, with each group having a fairly similar distribution of age and sex within the subgroups. Viral spread and transmission within the herd was facilitated during shared daytime pasture access and between some distinct breeds by night due to group housing with possible direct, nose-to-nose contacts or fomite transfer (feed racks and water troughs). Other aspects and measures of this outbreak were less fortunate for our retrospective data analysis. Fever is a key clinical sign of EHV-1 infection, and high fevers are typically a sign of viremia. Due to a lack of documentation other than “fevers noticed in various breeds (and stable units)” and “fevers in about one third of animals,” the assumption of an even spread of infection over the entire herd is questionable. Furthermore, although all animals, regardless of ownership, breed, age, or sex, received a single immune stimulant injection, and all animals with a fever, as long as the fever persisted, were treated with an NSAID, these measures did not interfere with our data analysis. As some animals were treated with dexamethasone, there may be risk of study bias. However, study bias is probably only minor as dexamethasone administration occurred in a small number (about 10) of horses/ponies with prolonged fever episodes and was not limited to members of a particular breed. 

However, the main findings of this outbreak were an increased risk for EHM in specific breeds, warmblood horses and Fjord horses, and, apparently, a decreased risk in small pony breeds, Shetland ponies and Welsh ponies. These findings are in further support of the risk factors previously suggested by Goehring et al. (2006) [2], which then did not reach statistical significance due to a small sample size. This group analyzed 9 EHM outbreaks involving a total of 195 horses and ponies on unvaccinated (EHV-1) premises in the Netherlands and suggested an increased risk for EHM in the Draft horse, Standardbred, and Hispanic breeds (PRE, Lipizzaner). In addition, EHM numbers in the Dutch study had also been extremely low in small pony breeds (Shetland and Welsh). However, their concerns were (partial) mitigation during an outbreak, different management, separate locations for some of the breeds on premises, and a small sample size. This is the first report suggesting an increased risk of developing EHM in Fjord horses. To our knowledge, and after screening the literature, there have not been reports of EHM outbreaks on (single-breed) Fjord, Icelandic, or Haflinger horse operations in EHV-1 endemic areas, while sizable operations exist as single-breed operations. Goehring et al. [2] have already suggested a reduced risk for EHM in small (archetypical-type) ponies during EHV-1 outbreaks and included Icelandic horses, Shetland and Welsh ponies, and also the Fjord and Haflinger horses in their discussion, mainly based on an assumed common ancestry and similar genetical background. However, our data from this outbreak suggest differently. It is worth pointing out that Fjord horses were group-housed by night, together with Welsh ponies, Icelandic horses, and other (mixed-breed) ponies, sharing the same air space and through direct contacts, increasing the likelihood of (smear) infection transmission. These intensified contacts extend to another group of horses that were stabled by night in the same building (stable C). This unit included all warmblood, warmblood–Arabian, and purebred Arabian horses. Only 1 out of 12 Arabian horses developed EHM, while almost half (10 animals) of the warmblood horses and interestingly 5 of the 12 Arabian–warmblood crossbred horses developed EHM. Our findings suggest a risk-enhancing factor for EHM in breeds such as warmblood and Fjord, while a protective factor or the absence of a factor apparently lowered the risk for EHM in the Arabian horse and various pony breeds. We made these observations among members of a particular line of Arabian horses, the Shagya Arabian, which has been shown to be genetically distinct from other Arabian horse lineages [13,14]. This could mean that our observations strictly relate to this particular lineage of Arabian horses. However, there are no reports of EHM outbreaks on any of the many renowned Arabian horse farms around the globe, including those in EHV-1 endemic areas, and Arabian horses with EHM have been underrepresented during mixed-breed operation outbreaks that differentiated between breeds. Genetic diversity also exists between various warmblood stud books, and as Welsh ponies are categorized into several subgroups (A–D), with each letter indicating a different size and phenotype, this should also hold true for the Welsh pony. During a genomewide association study, Brosnahan et al. found a single-nucleotide polymorphism (SNP) in an intron of the tetraspanin 9 gene, located on chromosome 6, when they compared horses with a fever and EHM with matching horses that had only developed a fever during experimental infection/natural EHV-1 outbreak conditions. As this study suffered from small numbers of horses to compare, it could be of importance to investigate and compare this particular SNP between warmblood, Fjord, Welsh, Shetland, and Arabian horses.

Age has been suggested to influence the risk for EHM development. These propositions are based on a controlled infection study where one group of female horses <15 years of age and another group >20 years of age were infected simultaneously. In group 1, only 1 out of 12 horses developed EHM, while 8 out of 12 horses did so in group 2 [1]. Two studies of naturally occurring EHM outbreaks claim more EHM cases in horses older than 3 years [2] or older than 5 years [4], with the first study suggesting an age cut-off between adolescence and adulthood. Our study population, with a mean age of about 18 years, was relatively old, with the youngest animal at 6 years of age. EHM cases were scattered among all age groups, and we did not observe a (linear) increase in EHM incidence with age. When we split the breeds into low-risk and high-risk breeds, it appeared that those few (*n* = 5) EHM cases in the low-risk group were all younger than 20 years of age. However, due to the coincidence of low numbers, “other factors,” not yet determined for this particular outbreak, could be responsible for these skewed numbers. Other factors could include previous exposure, co-infection, or other environmental factors simultaneously occurring in all age groups.

Traub-Dargatz et al. [5] observed that female horses developed EHM upon infection 4.3 times more often than male horses. Van Maanen et al. [12] found an almost equal distribution of EHM between male and female animals, although females were more often recumbent and thus more severely affected (similar to our EHM grade II). The multiple-outbreak study from the Netherlands came to similar conclusions [2], while we also did not detect a gender correlation here. The main difference between these reports is actually the environment or circumstances of the outbreak. Traub-Dargatz describes an outbreak during a (multiple-day) competition, where horses had been transported over long distances to attend. Van Maanen, Goehring, and our outbreak described here are reports from home premise outbreaks under presumably more stable horse boarding circumstances. However, these three reports describe outbreaks in one geographical region (central Europe) and all are outbreaks among unvaccinated (for EHV-1) animals. The results obtained by Traub-Dargatz et al. [5] originated from (partially) EHV-1-vaccinated animals. While finalizing this manuscript, we became witness to an extraordinary EHM outbreak among a sizable group of warmblood horses, originating from several different European countries, that were gathering in Valencia, Spain, for a multiple-week competitive/training event. Early reports described a relatively young (<15 years of age) horse population, and the group had been reported to be partially vaccinated against EHV-1. There are currently no confirmed data available of the male/female ratio of the horses attending this show; however, apparently, the majority of EHM-affected horses were female [15]. These early details resemble the situation described by Traub-Dargatz, with additional stressors of competition and travel, different from the circumstances described by others and us. While data collection on this outbreak is ongoing, it shows that most likely multiple factors, potentially also with different levels of impact, are necessary to turn an EHV-1 infection into EHM.

There exists an opinion among some horse owners, and even among some veterinarians, that vaccination against EHV-1 puts a horse at a greater risk of developing EHM upon EHV-1 infection than if it were unvaccinated. In our study, with a 23% EHM fatality rate in the entire (unvaccinated) herd, it can be safely said that vaccination against EHV-1 has not contributed to the risk of developing EHM during this outbreak. However, during the 2011 EHM outbreak among mainly (young) Paint horses in the US, described by Traub-Dargatz et al. [5], horses that were vaccinated against EHV-1 (and other diseases) within a 5-week period prior to EHV-1 exposure were, apparently, at an increased risk of developing EHM compared to those vaccinated more than 5 weeks ago. These findings could suggest a unique window of vulnerability for recently vaccinated horses, which needs further investigation.

In summary, EHM is most likely a multi-factorial disease following EHV-1 infection. Numerous factors could play a role in the pathogenesis, resulting in different levels of disease severity: virus strain variation, infectious dose and duration of exposure, host immunity, and other factors. The main findings of our study are that it seems likely that an increased risk for EHM exists in certain breeds over others, and within a high-risk breed, the influence of age seems less important. Furthermore, we could not identify an influence of sex. A meta-analysis of several outbreak data sets is necessary to strengthen a risk factor hypothesis; however, as each outbreak is uniquely different from the next in so many aspects, this will be a very difficult task. In the meantime, early detection of an index case with EHM, early identification of shedders through testing, and separating shedders from non-shedders will slow down disease spread and decrease EHM incidence regardless of “other factors”.

## 4. Materials and Methods

**Figure 3:**
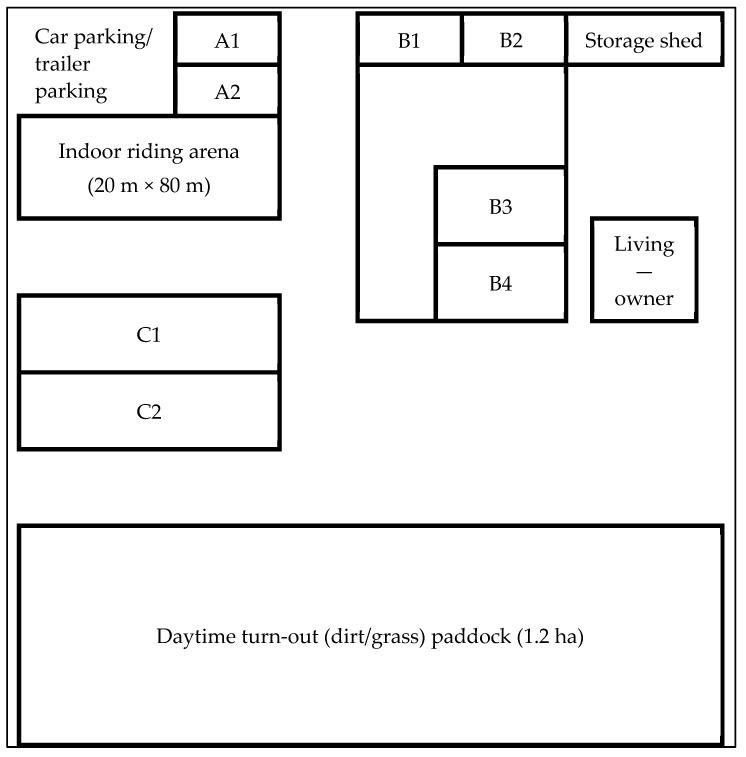
Schematic overview of the premises (not in scale) with commingling of all animals on daytime turn-out (dirt/grass) area, the indoor riding arena, a storage shed, the home of the owner, and with stable units A–C for night time housing.

This outbreak started during December 2016, and quarantine was lifted by the end of March 2017. Data were collected retrospectively during site visits and through phone interviews with the facility owner, veterinarians of the involved practices, and board members of the riding association, who were also boarders of horses at the facility at that time. Data collection started a little over a year after the outbreak and took about 6 months. The premises are located in rural southeastern Germany, at a 40 km distance from a larger city. Previously, the operation was used as a stud farm for (Shagya) Arabian horses and converted into a mixed-breed horse/pony operation during the early 2010s, offering boarding, a commercial riding program for children, and a retirement home for old horses. There were 143 horses/ponies on the grounds at the beginning of the outbreak. Two thirds of the herd was owned by the farm owner, and the remainder was individually owned (one person with 1 or 2 animals). The total surface of the operation encompasses about 12 ha. It includes several hectares of pasture (not used during the outbreak), a central 1.2 ha area of all-weather pasture, an indoor riding arena, three main stables (A–C), a storage building, and the living quarters (Figure 3).

Detailed information on (i) ID of the horse, (ii) age, and (iii) sex were available for the time point at the beginning of the outbreak. With the exception of a group of 12 “mid-size” ponies, a clear breed affiliation was available for each animal (131 animals). The defined breeds were (i) 21 Shetland ponies, (ii) 7 Icelandic horses, (iii) 22 Welsh ponies, (iv) 26 Fjord horses, (v) 12 (Shagya) Arabians, (vi) 12 Arabian–warmblood (F1) crossbred horses, and (vii) 23 warmblood horses (various stud books). Another 8 horses (1 Haflinger horse, 1 Haflinger–crossbred, 3 (American) Quarter horses, and 3 Draft horses), combined in a group of “others,” were excluded from breed-dependent calculation because of a small sample size. The youngest animal was 6, and the oldest 31 years old. Only castrated males (*n* = 69) and intact females (*n* = 74) were on the grounds (Figure 1a, Table 3). 

During daytime, all animals combined spent a minimum of 6 h on an all-weather pasture area (Figure 3). While outside, hay was supplied along the side closest to the farm buildings, and there was a single shared water source (trough). At night, the animals were housed in three separate stable buildings (A, B, C; Figure 3). Each building was divided into two to four subunits, as described. Fences or feeding (hay) racks were used as dividers. Uniformly, straw (pot stall system) was used for bedding. Shetland ponies were housed in A1 and A2. Stable B (B1, B2, B3, and B4, with a 2 m service aisle between B2 and B3) housed Fjord, Welsh, Icelandic, and Haflinger horses and the mid-size, yet unspecified, ponies. Stable C housed Arabian, warmblood, and Arabian–warmblood crossbred horses in two units (C1, C2) divided by a feed rack. Spread of horses/ponies over the subunit was at random, not by breed. Hayracks in a building were typically used by animals from both sides. Feed was the same for all stables consisting of a mixture of straw, hay, and alfalfa silage, as well as oats and barley. Only some of the privately owned horses were vaccinated (various products) against tetanus and equine influenza. One Icelandic horse (a 12-year-old gelding) was regularly (every 6 months) vaccinated against EHV-1 using a modified-live virus vaccine (Prevaccinol^®^, MSD Animal Health, Unterschleißheim, Germany). Detailed computed information included which animal developed EHM. The animals with the severe or tetraplegic form of EHM were euthanized within 24 h of non-improvement and categorized as EHM-II (severe), and marked in the notes accordingly. All horses/ponies with signs of ataxia, weakness, or urinary incontinence that remained standing were categorized as EHM-I (mild to moderate). Two animals were euthanized during the outbreak, but it was unclear whether they showed signs of colic or EHM-II. A necropsy was not done, and without information about the cause of recumbency, both a 21-year-old Welsh pony gelding and a 26-year-old warmblood mare were omitted of any calculation, bringing down the number from 143 to 141. Rectal temperature data were collected daily from all animals, fever was defined as a temperature >38.2 °C, and temperature data were not recorded. 

### Statistical Methods

We used Microsoft Office 2019 for data processing and management. For statistical analysis, R version 3.6.3 (2020-02-29 R Foundation for Statistical Computing, Vienna, Austria) statistical software was used. The graphic representation was done with Microsoft Office, R statistical software, and GraphPad Prism 5.04. Breed, gender, and EHM category were treated as categorical variables, and age as a continuous variable. The level of significance was pre-set as α ≤ 0.05. Descriptive statistics of different breeds and gender, including mean and median age, age range, standard deviation, interquartile range, and numbers of female vs. male, is available in Table 3. A multivariate Bayesian logistic regression without interactions was performed, with breed, gender, and age as independent variables, and EHM as a dependent variable. A Bayesian approach was chosen due to a small number of observations of some breeds, which then resulted in more reliable estimates of odds ratios and their confidence intervals compared to a frequentist generalized logistic regression. The association between independent variables was checked with the chi-square test for categorical variables and with Kruskal–Wallis and Mann–Whitney tests for age due to the non-normal distribution. The multicollinearity of independent variables was checked with variation inflation factor (VIF). There was no association between independent variables, and multicollinearity assumption was not violated (all VIF < 5) (Table 2). Due to the exploratory approach of the study, correction of the *p*-values for multiple comparisons during post hocs was not performed. We categorized animals according to breed. As only a few representatives of certain breeds were among the total, those were categorized as “others” and did not participate in evaluation (Draft, Quarter, Haflinger horse). Some of the ponies with unknown genetical background were categorized as “other pony” and were not evaluated as well. The breeds included in the calculation were (i) Shetland pony, (ii) Icelandic horse, (iii) Welsh pony, (iv) Fjord horse, (v) Arabian, (vi) Arabian–warmblood (F1), and (vii) warmblood. Sex categories were either male (castrated) or female (intact). The number of horses included for the multiple Bayesian logistic regression was 121.

## Figures and Tables

**Table 1:** EHM cases per breed, age, and sex.

Breed		*n*	Age					
			Min	Max	Mean	Median	SD *	IQR **
Warmblood horse		10	8	27	18.20	18.0	6.32	10.00
Arabian–WB crossbreed		5	9	18	13.40	13.0	3.65	5.00
Arabian		1	15	15	15.00	15.0	N/A	0.00
Fjord horse		13	10	29	17.31	18.0	5.86	7.00
Welsh pony		1	18	18	18.00	18.0	N/A	0.00
Icelandic horse		2	7	8	7.50	7.5	0.71	0.50
Shetland pony		1	13	13	13.00	13.0	N/A	0.00
Sex	Male	19	8	29	15.63	15.0	6.18	6.50
	Female	14	7	25	17.00	18.0	5.45	6.25

* SD: standard deviation; ** IQR: interquartile range.

**Table 2:** Results of the multiple Bayesian logistic regression (*n* = 121).

Predictors	Odds Ratios	CI	*p*-Value
(Intercept)	1.94	0.42–8.84	0.389
Shetland pony	0.09 *	0.02–0.55	0.008
Icelandic horse	0.46	0.09–2.37	0.348
Welsh pony	0.09 *	0.01–0.52	0.007
Arabian	0.19	0.03–1.16	0.069
Arabian–WB crossbreed	0.83	0.22–3.05	0.773
Warmblood	1.10	0.37–3.29	0.866
Age	0.94	0.88–1.02	0.121
Sex (male)	1.29	0.53–3.11	0.573

* marks the significant values.

**Table 3:** Herd details: distribution and specification.

Breed		*n*	Age						Female/Male
			Min	Max	Mean	Median	SD	IQR	
Shetland pony		21	10	28	19.86	24.0	7.53	14.00	10/11
Icelandic horse		7	7	21	14.14	13.0	5.61	9.00	3/4
Welsh pony		22	7	30	18.14	19.0	7.95	14.50	13/9
Fjord horse		26	6	29	17.04	18.0	6.24	9.75	13/13
Arabian		12	15	25	20.25	20.0	3.91	6.75	8/4
Arabian–WB crossbreed		12	6	26	15.83	14.5	6.31	8.00	6/6
Warmblood horse		23	8	27	19.22	20.0	5.32	8.00	9/14
Other ponies		12	6	26	16.83	17.5	5.98	7.00	8/4
Other horses		8	13	31	20.62	20.5	5.88	6.50	4/4
Sex	Male	69	6	31	18.58	19.0	6.82	12.00	0/69
	Female	74	6	29	17.81	18.5	6.11	10.00	74/0
Total		143	6	31	18.18	19.0	6.45	11.00	74/69

## Data Availability

The data presented in this study are available on request from the corresponding author.
